# Type 2 Diabetes Mellitus in Nepal from 2000 to 2020: A systematic review and meta-analysis

**DOI:** 10.12688/f1000research.53970.2

**Published:** 2021-09-06

**Authors:** Dhan Bahadur Shrestha, Pravash Budhathoki, Yub Raj Sedhai, Achyut Marahatta, Samit Lamichhane, Sarbin Nepal, Anurag Adhikari, Ayusha Poudel, Samata Nepal, Alok Atreya

**Affiliations:** 1 Department of Emergency Medicine, Mangalbare Hospital, Morang, 56600, Nepal; 2 Department of Emergency Medicine, Dr. Iwamura Memorial Hospital, Bhaktapur, 44800, Nepal; 3 Department of Internal Medicine, Virginia Commonwealth University, School of Medicine, Richmond, Virginia, 23298, USA; 4 Chitwan Medical College Teaching Hospital, Chitwan, 44200, Nepal; 5 Department of Emergency Medicine, Nepal National Hospital, Kathmandu, Bagmati, 44600, Nepal; 6 Department of Emergency Medicine, Alka Hospital, Lalitpur, Bagmati, 44600, Nepal; 7 Department of Community Medicine, Lumbini Medical College, Palpa, Lumbini, 32500, Nepal; 8 Department of Forensic Medicine, Lumbini Medical College, Palpa, Lumbini, 32500, Nepal

**Keywords:** Blood Pressure, Body Mass Index, Diabetes Mellitus Type 2, Nepal

## Abstract

**Aims:** To evaluate the prevalence and risk factors of type 2 diabetes mellitus (T2DM) from 2000-2020 in various parts of Nepal. **Methods:** PubMed, Embase, Scopus, and Google Scholar were searched using the appropriate keywords. All Nepalese studies mentioning the prevalence of T2DM and/or details such as risk factors were included. Studies were screened using Covidence. Two reviewers independently selected studies based on the inclusion criteria. Meta-analysis was conducted using Comprehensive Meta-Analysis Software v.3. **Results:** A total of 15 studies met the inclusion criteria. The prevalence of T2DM, pre-diabetes, and impaired glucose tolerance in Nepal in the last two decades was 10% (CI, 7.1%- 13.9%), 19.4% (CI, 11.2%- 31.3%), and 11.0% (CI, 4.3%- 25.4%) respectively. The prevalence of T2DM in the year 2010-15 was 7.75% (CI, 3.67-15.61), and it increased to 11.24% between 2015-2020 (CI, 7.89-15.77). There were 2.19 times higher odds of having T2DM if the body mass index was ≥24.9 kg/m ^2^. Analysis showed normal waist circumference, normal blood pressure, and no history of T2DM in a family has 64.1%, 62.1%, and 67.3% lower odds of having T2DM, respectively. **Conclusion:** The prevalence of T2DM, pre-diabetes, and impaired glucose tolerance in Nepal was estimated to be 10%, 19.4%, and 11% respectively.

## Introduction

The burden of type 2 diabetes mellitus (T2DM) is increasing across the globe with time. The growing prevalence of diabetes lead to big impact in societal socio-economic and health aspect ^1^. In 2019, the International Diabetes Federation (IDF) estimated that 463 million adults worldwide had diabetes ^1^. The statistics showed that these individuals were in the age range of 20 to 79, have diabetes, and 79.4% were from low- and middle-income countries ^1^. Additionally, IDF estimates that the global prevalence of diabetes will be 578.4 million by 2030, with this rising to 700.2 million by 2045 among adults aged between 20 to 79 years ^1^ In the region of Southeast Asia, the prevalence of diabetes was 8.8% in 2019, and this is projected to increase to 9.7% by 2030 ^2^. T2DM still remains a major cause of worldwide morbidity and mortality, which leads to complications such as neuropathy, nephropathy, stroke, and coronary artery disease ^3^. In 2017, over 10, 000 individuals died due to T2DM or diabetes-related complications in Nepal, which is the 11th most common cause of disability in terms of disability-adjusted life years (DALYs) (1226 DALYs per 10,000 population) ^4^. In 2020, the prevalence of T2DM in Nepal was 8.5% (95% CI 6.9–10.4%), which was higher than that of 8.4% (95% CI 6.2–10.5%) in 2014 ^5, 6^. Similarly, in 2020 the prevalence of pre-diabetes was 9.2% (95% CI 6.6 – 12.6%) compared to 2014, which was 10.3% (95% CI 6.1–14.4%) ^5, 6^. In the advent of growing non-communicable diseases, a Multi-Sectoral Action Plan has been adopted by the government of Nepal to prevent and control non-communicable diseases including T2DM ^7^. There are several prevalence studies across the countries and in different localities, however, there is no appropriate pooling of the data on the risk factors of T2DM in Nepal. Thus, with the objective to pool the available data on prevalence and risk factors for pre-diabetes, and T2DM in Nepal over the past 20 years we conducted this meta-analysis. Pooling the studies done in various parts of the country by gathering data of individual prevalence and risk factor study on T2DM can be helpful for the further prevention and control of this disease. This will build the foundation of the evidence for evidence based practice. 

## Methods

### Protocol registration

The systematic review is registered in PROSPERO (CRD42020215247). It is documented as per the guidelines of the Meta-Analysis of Observational Studies in Epidemiology (MOOSE) ^8, 9^.

### Information sources and search strategy

Electronic databases such as PubMed, PubMed Central, Google Scholar, Scopus, and Embase were used to search relevant articles ( Extended data file 1 ^10^). Published articles from 2000 to 2020 were searched with the use of the appropriate keywords such as “diabetes mellitus”, “high blood sugar”, “type 2 diabetes”, “prevalence”, “risk factor” and “Nepal” along with relevant Boolean operators.

### Eligibility criteria

All published studies that took place in Nepal from 2000–2020 were included in this review. These studies comprised of cross-sectional studies, case series that reported on more than 50 patients, cohort study, randomized control trial (RCTs) that were based on prevalence of T2DM and/or its related issues such as risk factors, outcome, and outcome predictors.

Editorials, commentaries, viewpoint articles without adequate data on T2DM and its related issues were excluded. Furthermore, studies that took place before 1999, outside of Nepal, as well as those that were on Type 1 and gestational diabetes were excluded. 

### Study selection

The studies were selected with the use of Covidence ^11^. The title and abstract were screened based on the inclusion criteria independently by two authors (SL, SN). Discrepancies were resolved by consensus obtained from the third author (AM). Further full-text review (SN, AM) was done independently, and discrepancies (SL) were resolved.

### Data collection process and data extraction

Three authors (SL, AM, and SN) were independently involved in the data extraction and adding that to a standardized form in Excel. The accuracy and completion of each other's work was verified by all the reviewers. The characteristics extracted for each selected study included, first author, year, study design, sample size, study location, prevalence rate, and risk factors of T2DM such as Body Mass Index (BMI), exercise (moderate to high level of exercise (≥ 30 minutes/days) is taken as adequate), waist circumference (≥85 cm in females, and ≥90 cm in males were defined as high), family history, fruit and vegetable serving per day, alcohol, smoking/tobacco, literacy, and increased blood pressure (BP) (≥140/90 mmHg is taken as hypertensive) (Please see *Underlying data* ^12^).

### Data analysis

Comprehensive Meta-Analysis Software (CMA) v.3 was used to analyze the extracted data.

### Definition of the condition

 T2DM was defined as a fasting blood glucose (FBG) of ≥ 126 mg/dl (7.0 mmol/l) or a 2-h oral glucose tolerance test (OGTT) blood glucose level of ≥ 200 mg/dl (11.1 mmol/l). Prediabetes was defined as FBG level between 100 (5.6 mmol/l) and 125 mg/dL (< 7 mmol/l) or a 2-h OGTT blood glucose level between 140 (7.8 mmol/l) and 199 mg/dl (11 mmol/l). Impaired glucose tolerance (IGT) was defined as two-hour glucose levels of 140 to 199 mg per dL (7.8 to 11.0 mmol) on the 75-g oral glucose tolerance test ^13^.

### Bias assessment

Bias assessment of the included studies was done by the Joanna Briggs Institute (JBI) tool ( Table 1) ^14^.

**Table 1.** JBI checklist for bias assessment.

Author/year	Was the sample frame appropriate to address the target population?	Were study participants sampled in an appropriate way?	Was the sample size adequate?	Were the study subjects and the setting described in detail?	Was the data analysis conducted with sufficient coverage of the identified sample?	Were valid methods used for the identification of the condition?	Was the condition measured in a standard, reliable way for all participants?	Was there appropriate statistical analysis?	Was the response rate adequate, and if not, was the low response rate managed appropriately?
Sharma B ^16^ *et al*. 2019	yes	yes	No	Yes	Yes	yes	yes	yes	yes
Gyawali B ^17^ *et al*. 2018	yes	yes	Yes	Yes	Yes	yes	yes	yes	yes
Sharma SK ^18^ *et al*. 2011	yes	yes	Yes	Yes	Yes	yes	yes	yes	yes
Sharma SK ^19^ *et al*. 2013	yes	yes	Yes	Yes	yes	yes	yes	yes	yes
Chhetri MR ^20^ *et al*. 2009	yes	yes	Yes	Yes	yes	yes	yes	yes	yes
Paudyal G ^21^ *et al*. 2008	yes	yes	Yes	Yes	yes	yes	yes	yes	yes
Bhandari GP ^22^ *et al*. 2014	yes	yes	Yes	Yes	yes	yes	yes	no	yes
Karki P ^23^ *et al*. 2000	yes	yes	Yes	Yes	yes	yes	yes	no	yes
Paudel S ^24^ *et al*. 2020	yes	yes	Yes	Yes	yes	yes	yes	yes	yes
Koirala S ^25^ *et al*. 2018	yes	yes	yes	Yes	yes	yes	yes	yes	yes
Ranabhat K ^26^ *et al*. 2016	no	yes	no	Yes	yes	yes	yes	yes	yes
Mehta KD ^27^ *et al*. 2011	yes	yes	yes	Yes	yes	yes	yes	yes	yes
Shrestha UK ^28^ *et al*. 2006	yes	yes	yes	Yes	yes	yes	yes	yes	yes
Dhimal M ^29^ *et al*. 2019	yes	no	yes	Yes	yes	yes	yes	yes	yes
Kushwaha A ^30^ *et al*. 2020	no	yes	no	Yes	yes	yes	yes	yes	yes

### Assessment of heterogeneity

The heterogeneity in the included studies was assessed based on the Cochrane Handbook for Systematic reviews by the I ^2 ^statistics (I ^2^>50%) ^15^. Thus, a random-effects model with the inverse variance heterogeneity model was performed. If I²>50% significant heterogeneity random effect model was preferred. If I²<50% then fixed effect model was preferred.

### Sensitivity analysis

The sensitivity analysis was performed by excluding studies that did not show any significant difference in the prevalence of T2DM.

## Result

**Figure 1.**
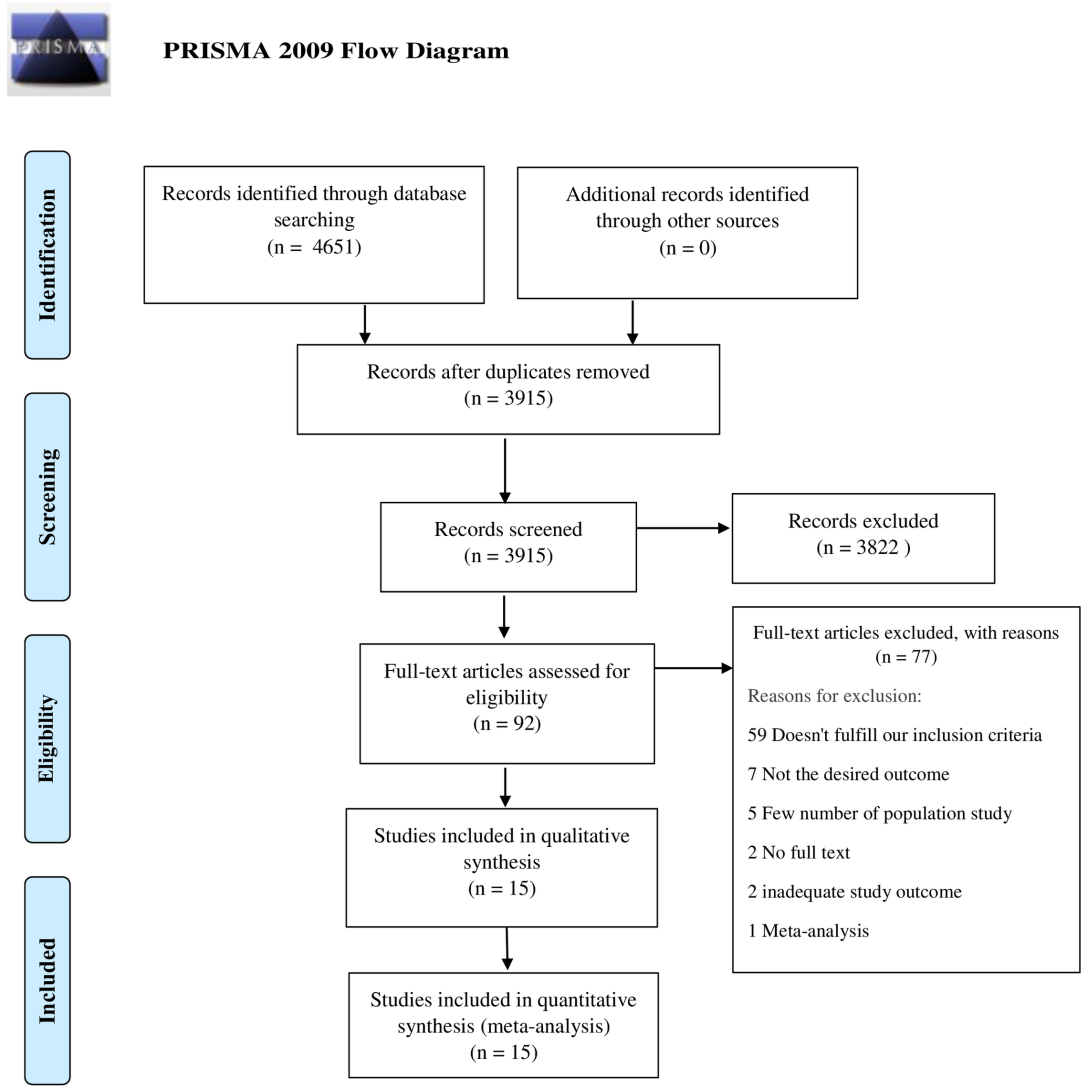
PRISMA flow diagram.

A total of 4651 studies were analyzed after thorough database search, of which 736 were identified as duplicates and removed. Title and abstracts of 3915 studies were screened and 3822 studies were excluded. The full-text eligibility of 92 studies was assessed and 77 studies were excluded for definite reasons. A total of 15 studies were included in the qualitative and quantitative analysis. The following information is depicted in the PRISMA flow diagram ( Figure 1).

### Qualitative summary

A qualitative summary of all 15 individual study is presented in ( Table 2). Nine studies were done in community setting while rest six were done in hospital setting. Two community based studies, and one hospital based study included sample from different part of Nepal to represent the country, while rest were loco-regional studies.

**Table 2.** Qualitative summary.

Author/s	Study Year	Study Design	Sample Size	Study Area	Pre-diabetes	T2DM	IGT
Dhimal M ^29^ *et al*. 2019	2019	Cross-sectional study	12557	72 districts (all provinces)		1067/12557	
Shrestha UK ^28^ *et al*. 2006	2006	Cross-sectional study	1012	Seven wards of metropolitan and sub-metropolitan of Nepal		192/1012	107/1012
Kushwaha A ^30^ *et al*. 2020	2020	Cross-sectional study	114	Community Hospital		5/114	
Sharma B ^16^ *et al*. 2019	2019	Cross-sectional study	320	Morang	55/320	38/320	57/320
Gyawali B ^17^ *et al*. 2018	2018	Cross-sectional study	2310	Lekhnath municipality	302/2310	271/2310	
Sharma SK ^18^ *et al*. 2011	2011	Cross-sectional study	14425	Eastern Nepal		889/14008	
Sharma SK ^19^ *et al*. 2013	2013	Cross-sectional study	3218	Dharan		242/3218	
Chhetri MR ^20^ *et al*. 2009	2009	Cross-sectional study	1633	Kathmandu valley		422/1633	
Paudyal G ^21^ *et al*. 2008	2008	Cross-sectional study	1475	Mulpani ,Gothar Kathmandu valley		60/1475	34/1475
Bhandari G ^22^ *et al*. 2014	2014	Cross-sectional study	11901	31 selected hospital institutions (28 non-speciality and 3 speciality)		391/11901	
Karki P ^23^ *et al*. 2000	2000	Cross-sectional Study	1,840	Outpatient clinic of BPKIHS		116/1840	
Paudel S ^24^ *et al*. 2020	2020	Secondary analysis of the data	1977	Across Nepal		179/1977	
Koirala S ^25^ *et al*. 2018	2018	Cross-sectional study	188(85M/103F)	Mustang district	59/188	9/188	
Ranabhat K ^26^ *et al*. 2016	2016	Cross-sectional study	154 (80M/74F)	Tribhuwan University Teaching Hospital of Nepal		66/154	
Mehta KD ^27^ *et al*. 2011	2011	Cross-sectional study	2006(1096M/910F)	Sunsari , Eastern Nepal		422/2006	80/289

BPKIHS, B.P. Koirala Institute of Health Sciences; F, female; IGT, Impaired Glucose Test; M, Male.

### Quantitative synthesis

A total of 15 studies were included in the quantitative analysis.

### Prevalence of T2DM

**Figure 2.**
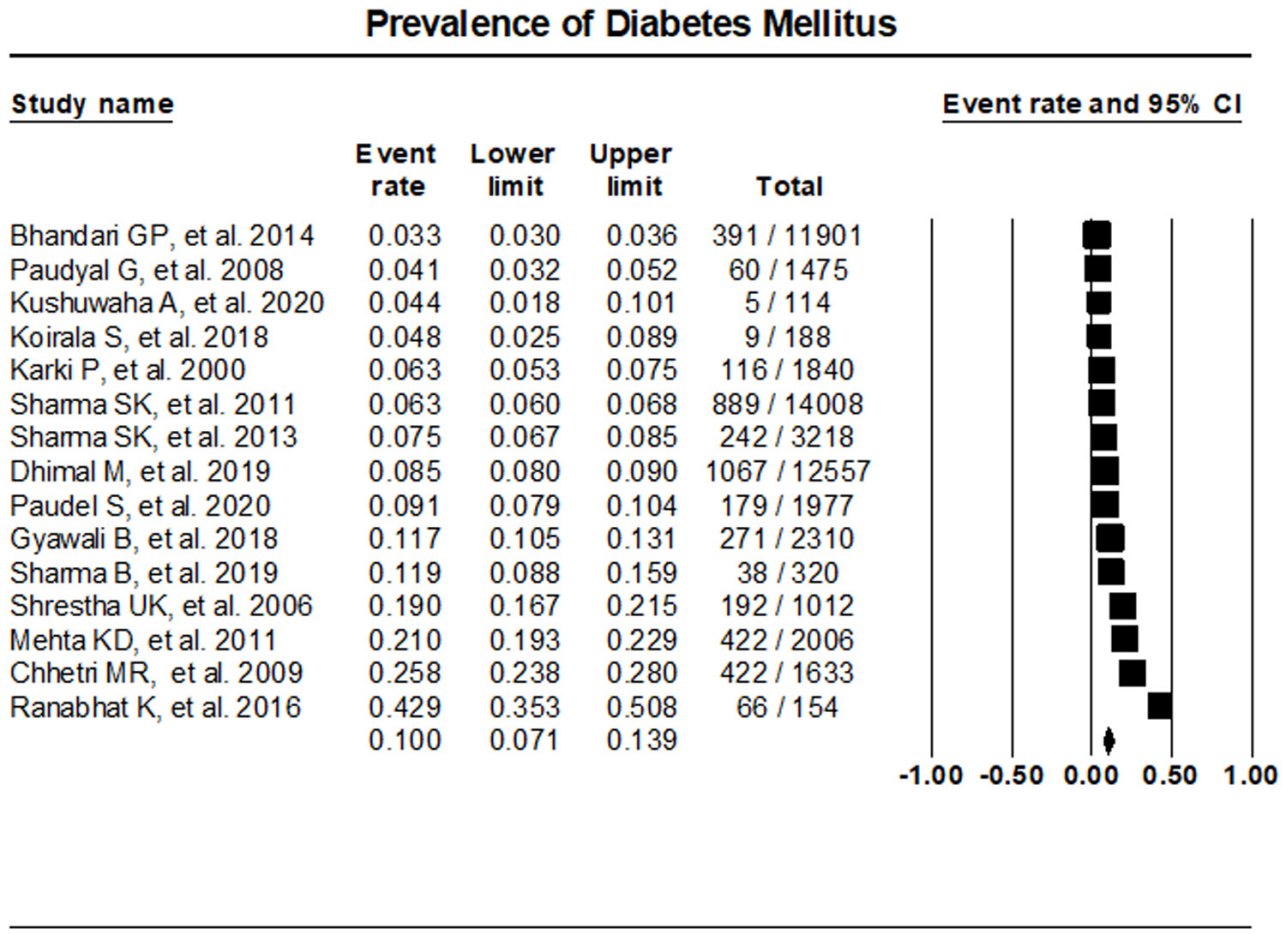
Prevalence of T2DM in Nepal.

The random effects meta-analysis assessment of 15 studies indicated T2DM prevalence at 10% (95% CI, 7.1%- 13.9%) ( Figure 2). Sensitivity analysis was performed with the exclusion of individual studies which resulted in no significant differences in the prevalence of T2DM ( *Extended data* file 2, Figure 1 ^10^)

**Figure 3.**
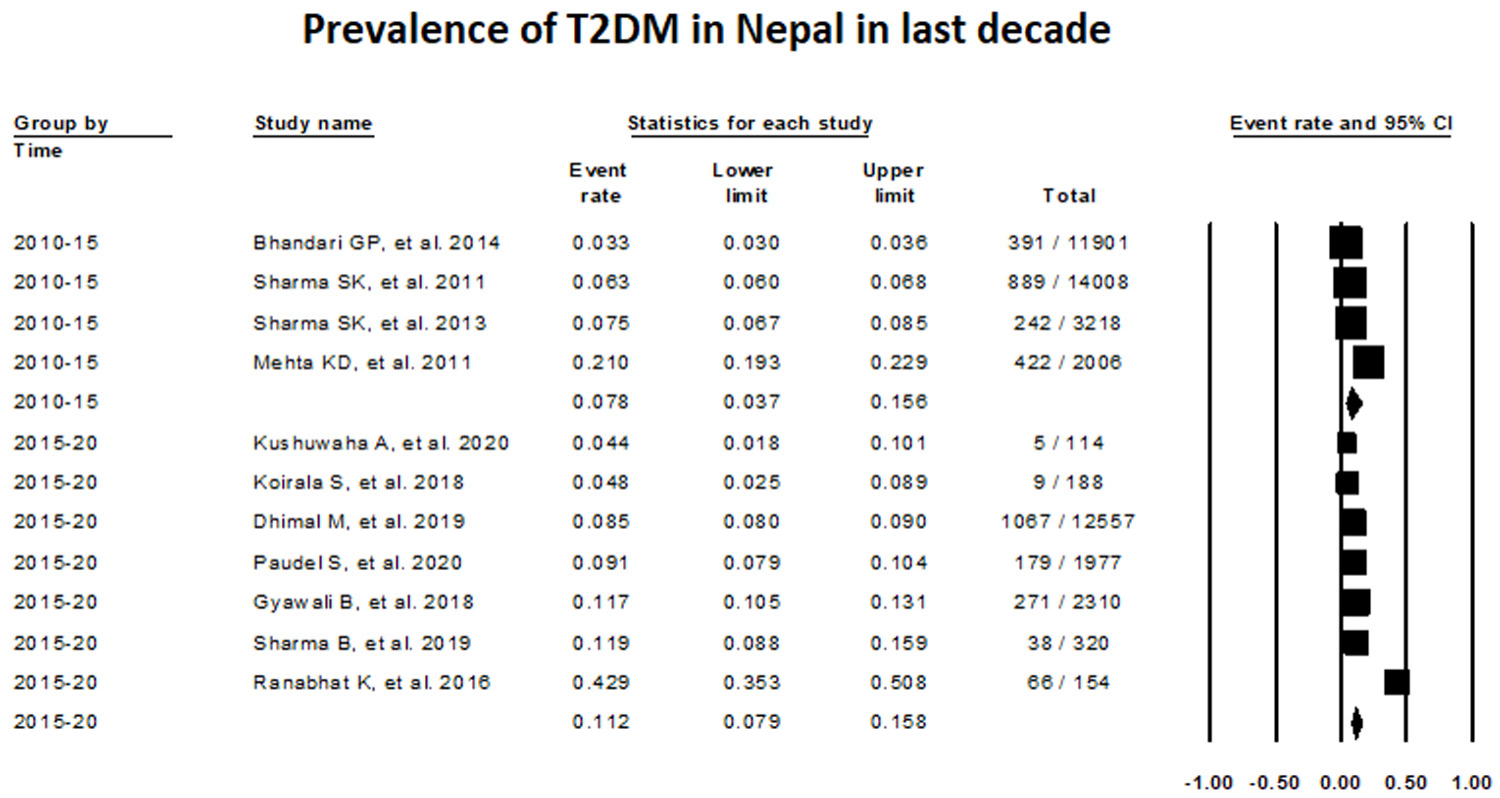
Prevalence of T2DM in Nepal taking consideration of time frame from 2010–2020.

The assessment of T2DM prevalence between 2010–2015 with the use of random-effects meta-analysis was 7.75% (Proportion, 0.0775; 95% CI, 0.0367-0.1561; studies: 4; I ^2^:99.62), while this value increased to 11.24%, between 2015–2020 (Proportion, 0.1124; 95% CI, 0.0789-0.1577; I ^2^: 96.74) ( Figure 3).

In relation to the study setting, the re-analysis of the data with the use of the random-effects model showed that 10.4% among surveyed adult population based on community-based studies had T2DM (Proportion, 0.1040; 95% CI, 0.0668-0.1596) ( *Extended data* file 2, Figure 2), while 9.23% among hospital/Directly observed, treatment short-course (DOTS) center-based studies have this disease (Proportion, 0.0923; 95% CI, 0.0509-0.1617) ( *Extended data* file 2, Figure 3 ^10^).

**Figure 4.**
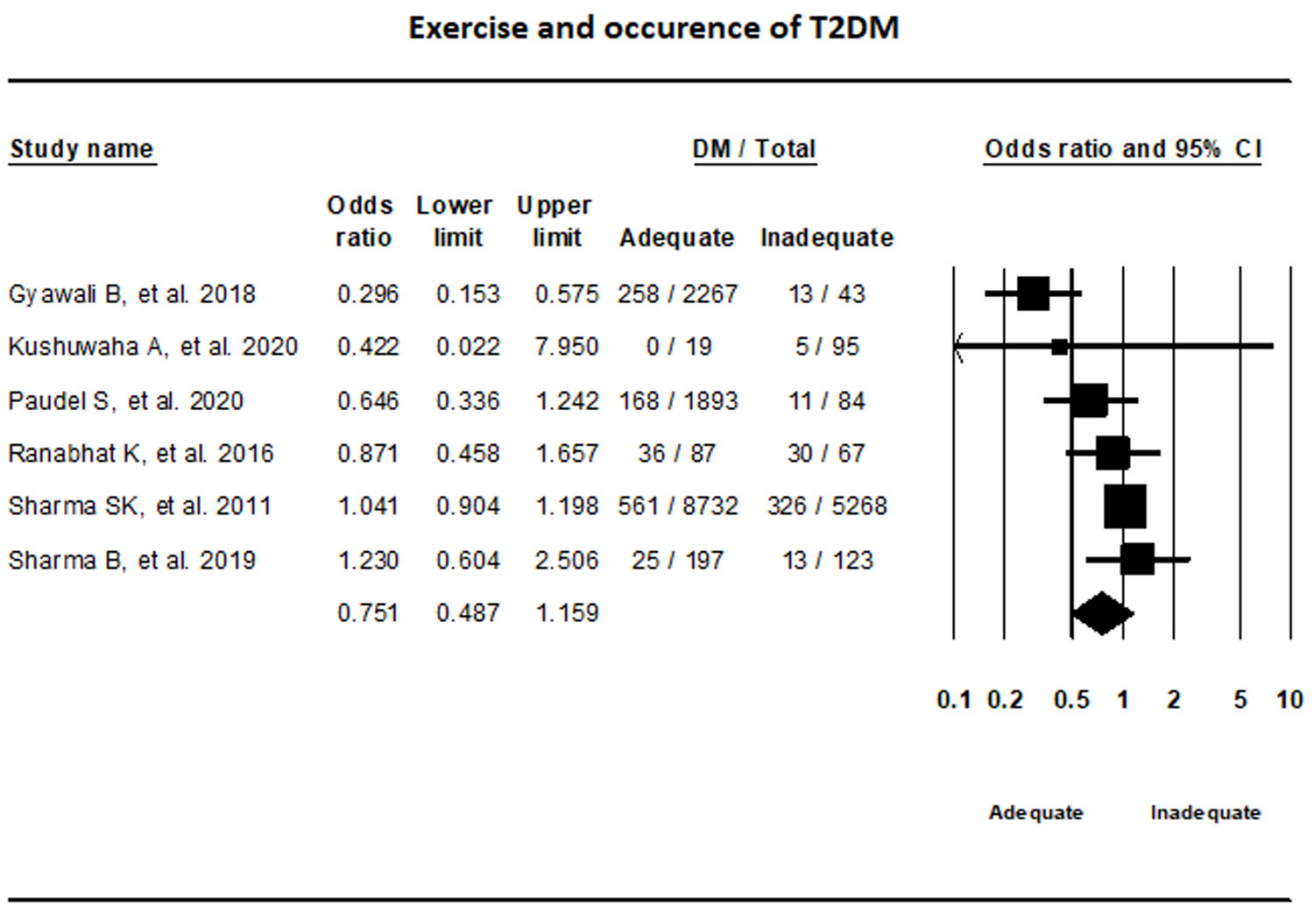
Forest plot showing exercise status and T2DM in Nepal.

**Figure 5.**
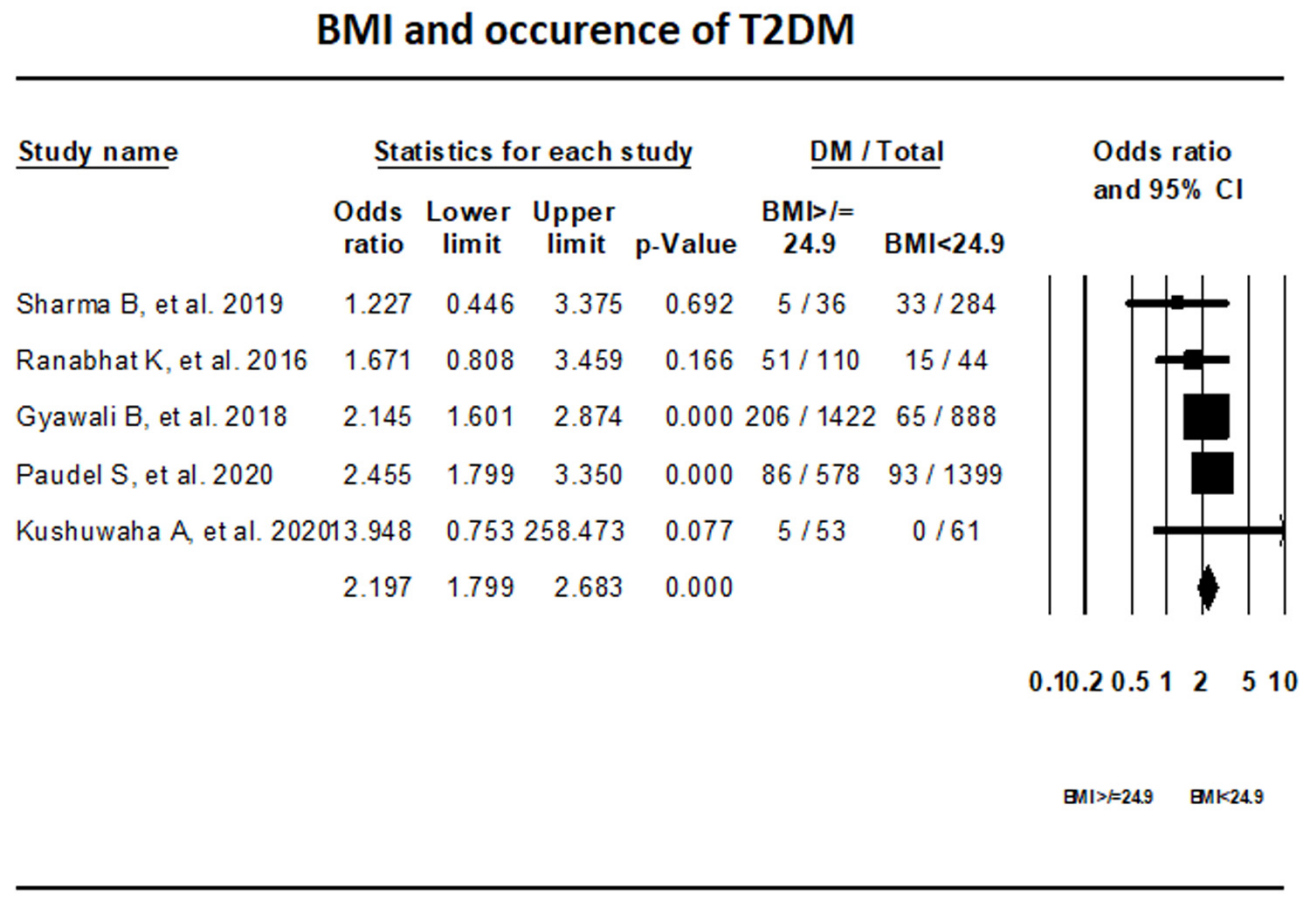
Forest plot showing BMI category and T2DM in Nepal.

Pre-diabetes was present in 19.4% (Proportion, 0.194; 95% CI, 11.2%- 31.3%) ( *Extended data* file 2, Figure 4) and IGT in 11.0% (Proportion, 0.110; 95% CI, 4.3%- 25.4%) ( *Extended data* file 2, Figure 5 ^10^).

### Risk factors of T2DM

***Exercise.*** Random-effects model that incorporated data from six studies on exercise showed that the difference in T2DM status between adequate and inadequate exercise groups were not statically significant (OR, 0.75, 95% CI, 0.49-1.16; I ^2^, 67.85%) ( Figure 4).

***BMI.*** Fixed-effect meta-analysis of five studies that reported on the BMI indicated that with a BMI ≥24.9 kg/m ^2 ^the odds of having T2DM is 2.19 times higher than with BMI <24.9 kg/m ^2^ (OR, 2.197; 95% CI, 1.799-2.683) ( Figure 5).

***Waist circumference.*** Individuals with healthy waist circumference had 64.1% lower odds of having T2DM compared with those with high waist circumference (OR, 0.361; 95% CI, 0.284-0.460; I ^2^, 0%) ( *Extended data* file 2, Figure 4 ^10^).

**Figure 6.**
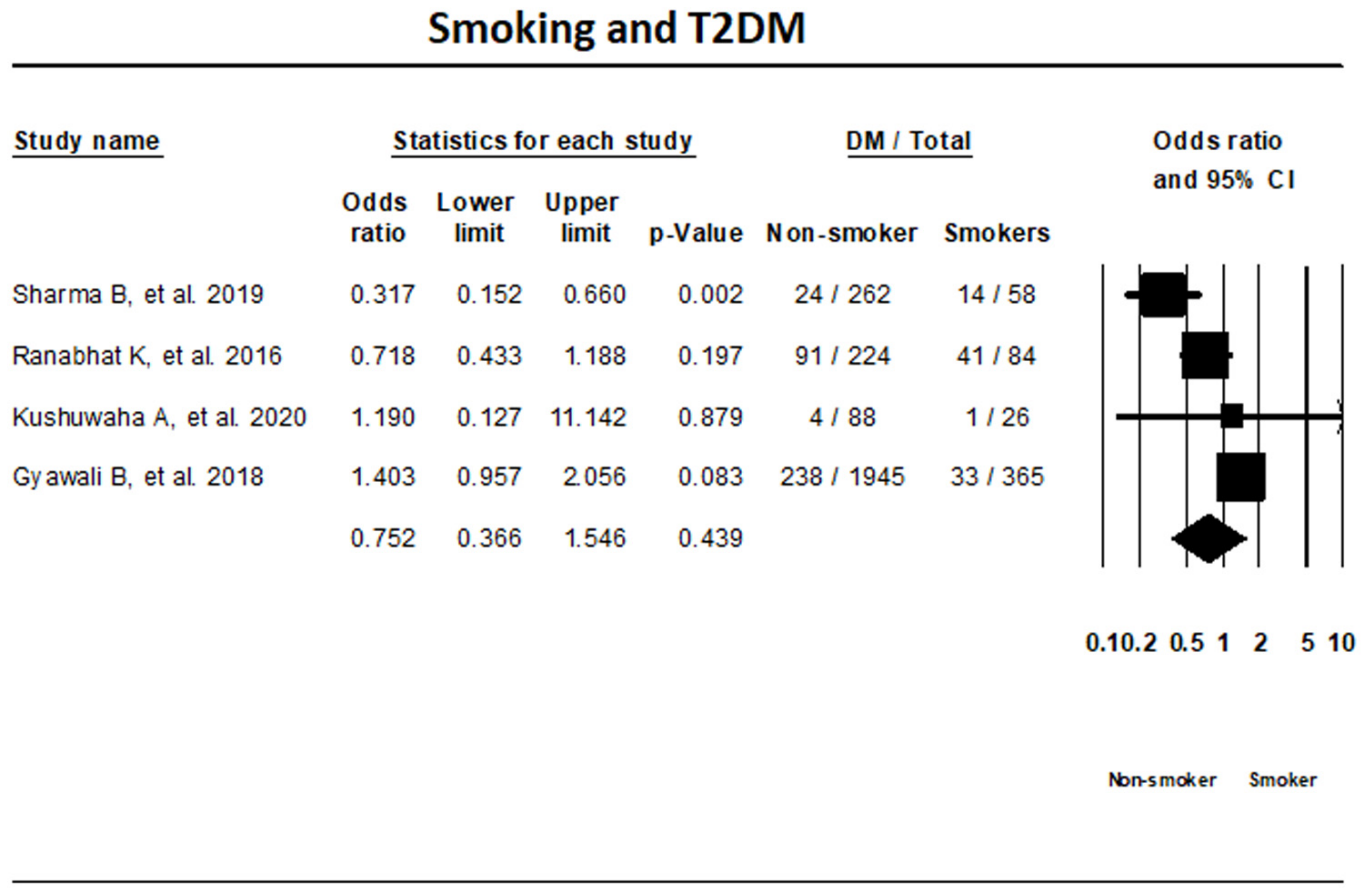
Forest plot showing smoking status and T2DM in Nepal.

***Smoking status.*** The random-effects meta-analysis of four T2DM studies based on smoking status indicated that the differences in T2DM status among smokers and non-smoker were not significant (OR, 0.752; 95% CI, 0.366-1.546; I ^2^; 87.2%) ( Figure 6).

**Figure 7.**
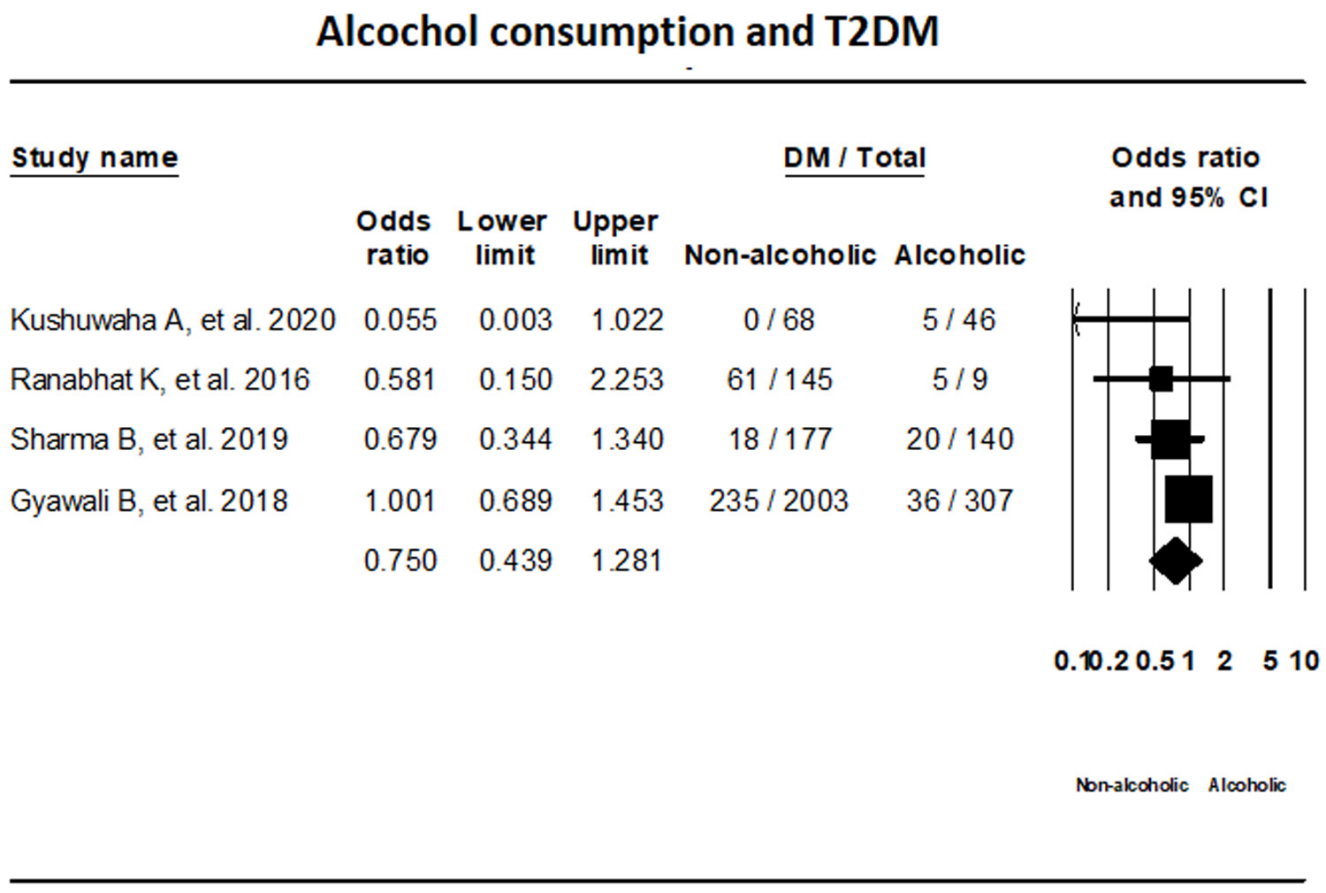
Forest plot showing alcohol consumption status and T2DM in Nepal.

***Alcohol consumption.*** T2DM status in relation with alcohol consumption was assessed by four studies with the use of random-effects model. The results showed that T2DM status among alcoholic and non-alcoholic groups were not statistically significant (OR, 0.750; 95% CI, 0.439-1.281 I ^2^; 37.72%) ( Figure 7).

**Figure 8.**
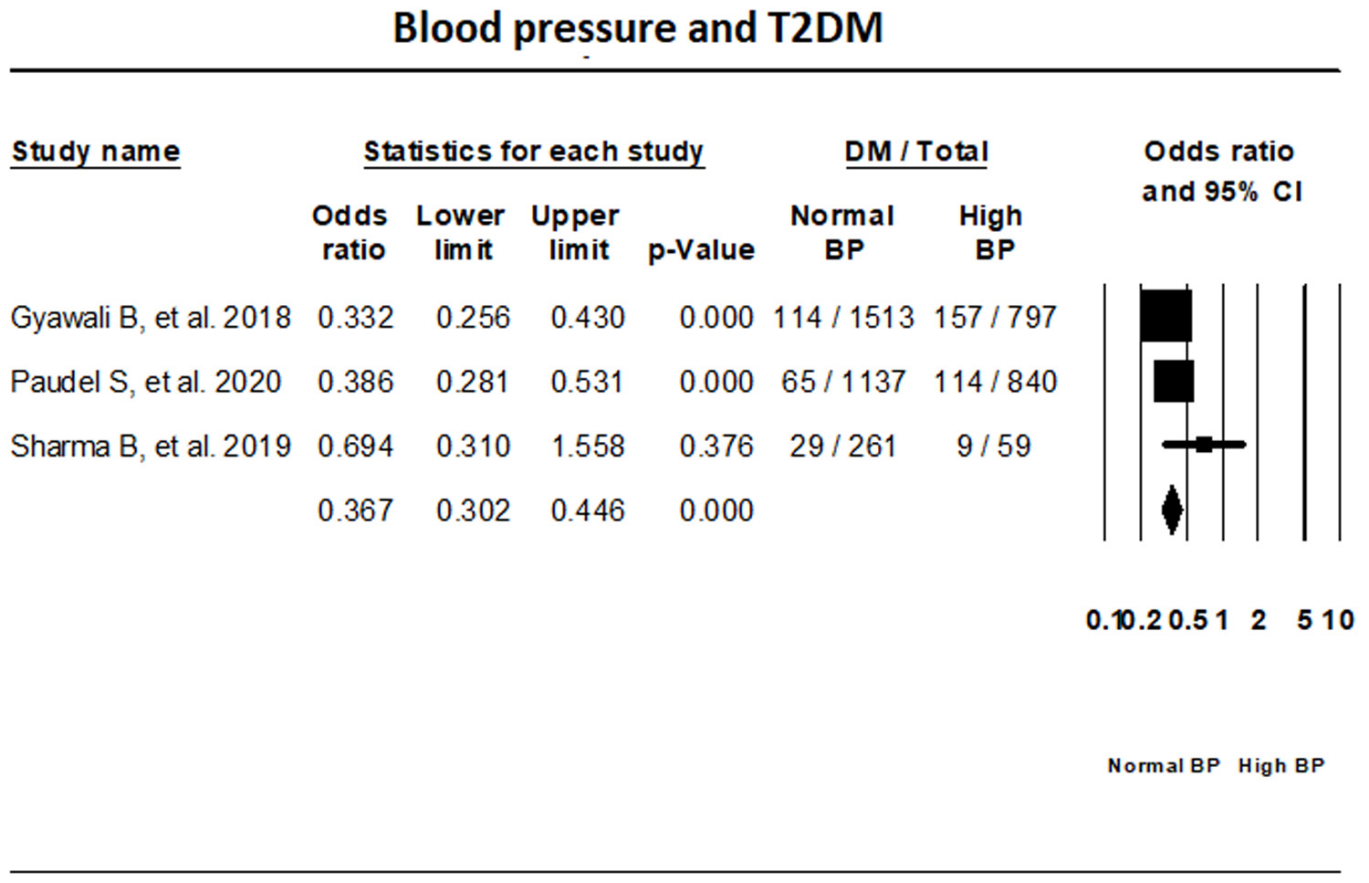
Forest plot showing blood pressure status and T2DM in Nepal.

***BP.*** Fixed-effect meta-analysis of three studies that have reported on T2DM status in relation with BP has indicated that the odds of individuals with normal BP having T2DM is 62.1% lower than those with high BP (OR, 0.379; 95% CI, 0.290-0.495) ( Figure 8).

**Figure 9.**
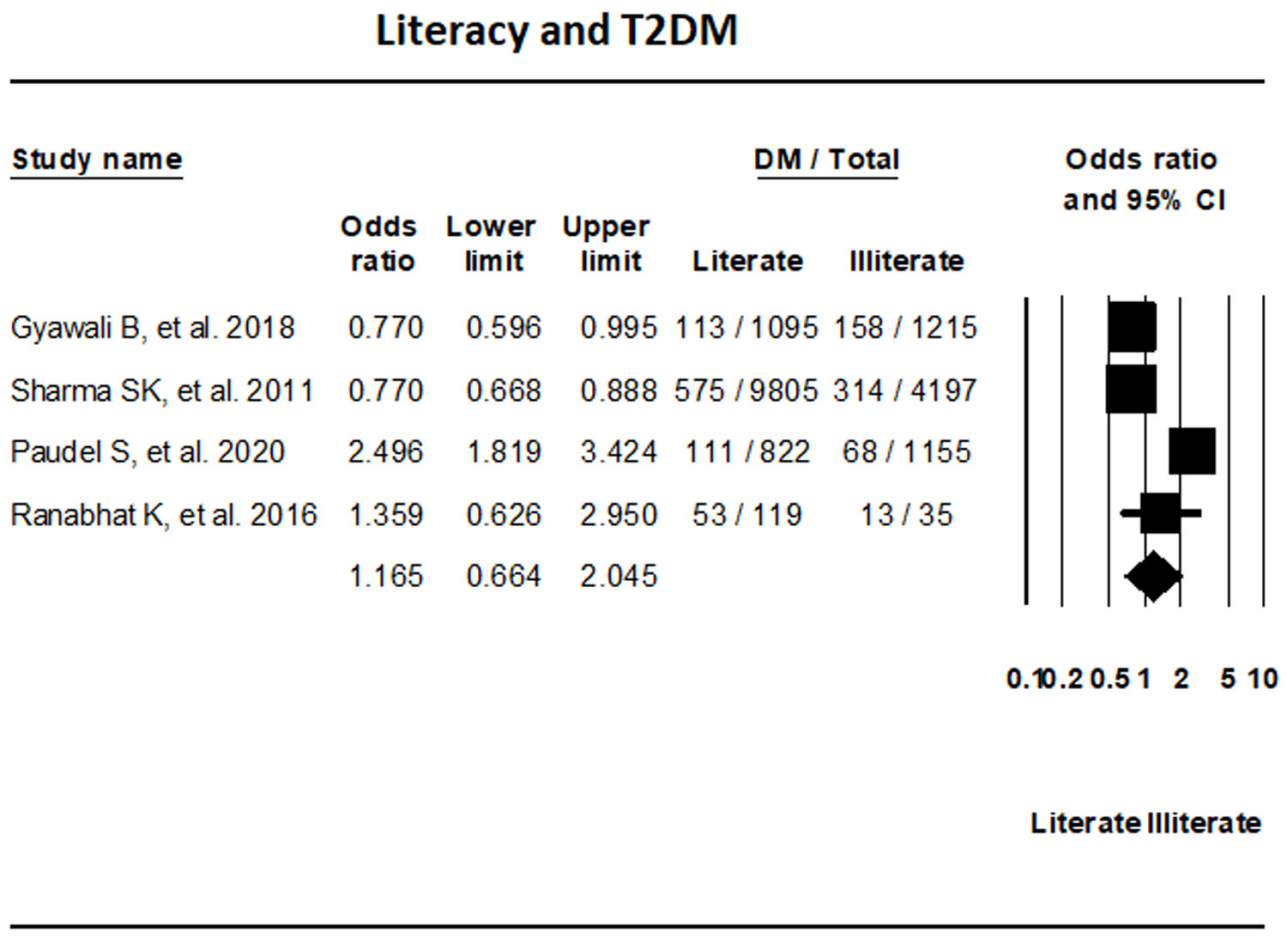
Forest plot showing literacy status and T2DM in Nepal.

***Literacy.*** The assessment of four studies that reported on T2DM based on literacy status did not show any significant differences in T2DM between literate and illiterate groups (OR, 1.165; 95% CI, 0.664-2.045; I ^2^, 93.61%) ( Figure 9).

**Figure 10.**
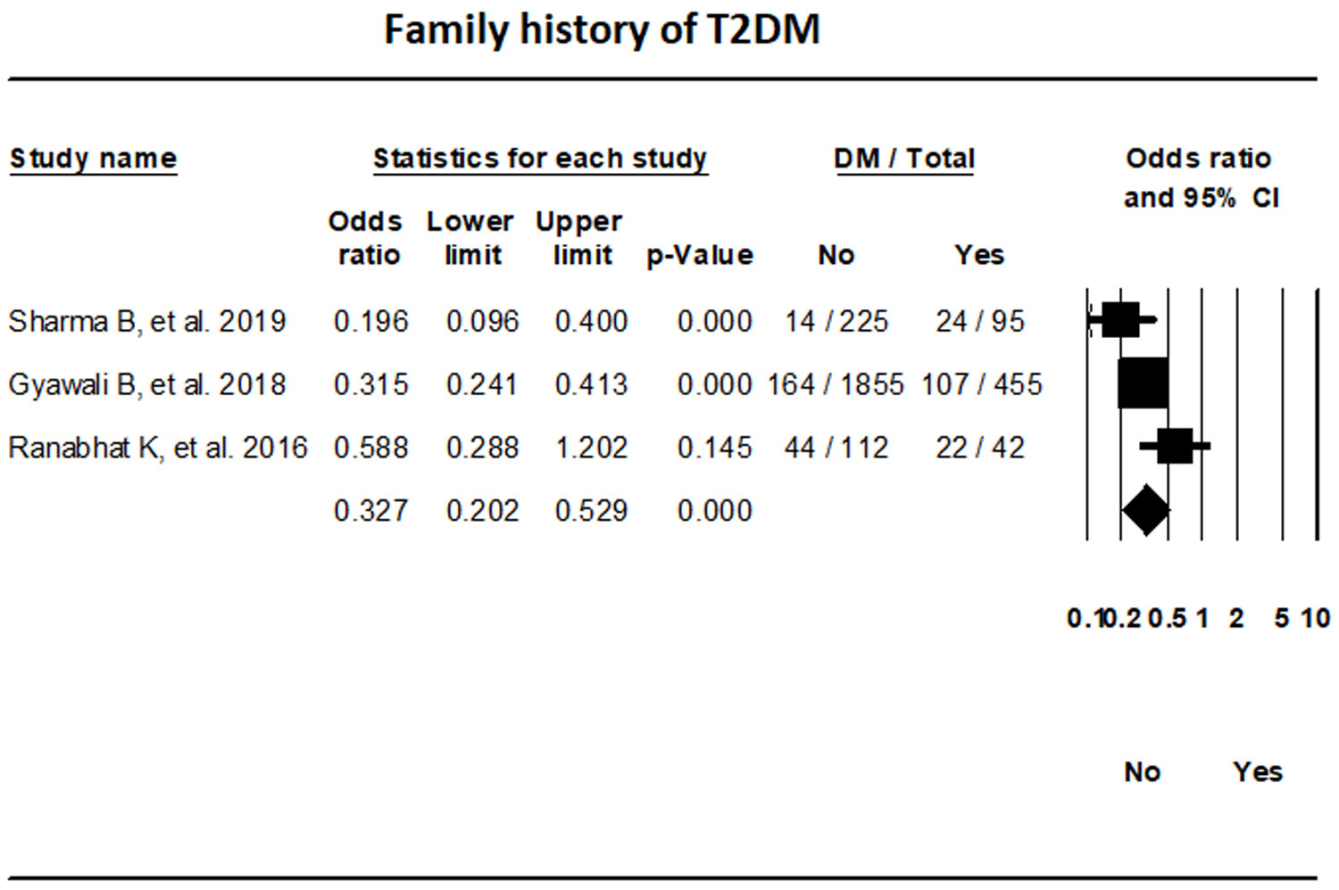
Forest plot showing the family history of T2DM and Diabetes status in patients in Nepal.

***Family history.*** The random-effects meta-analysis of three studies indicated that the odds of T2DM in individuals without a family history of T2DM were 67.3% lower in comparison to those with a family history (OR, 0.327; 95% CI, 0.202-0.529; I ^2^, 56.62%) ( Figure 10).

***Fruits and vegetables intake.*** The data assessment of the two studies that had reported on T2DM status in relation to fruits and vegetable intake did not reach a significant difference (OR, 0.933; 95% CI, 0.441-1.976; I ^2^, 78.72%). ( *Extended data* file 2, Figure 7 ^10^).

***Publication bias.*** Publication bias among the included studies were tested with the use of Egger’s test and was presented in a Funnel plot. The prevalence of T2DM in the Funnel plot showed an asymmetric distribution of studies, which suggested publication bias ( *Extended data* file 2, Figure 8 ^10^).

## Discussion

The prevalence of T2DM, pre-diabetes, and IGT in Nepal was found to be 10%, 19.4%, and 11% respectively. Our results show that in Nepal obesity is the highest risk factor for T2DM, while individuals with normal waist circumference and lack of family history of T2DM had lower risk of T2DM.

The estimated prevalence of T2DM was higher than that reported in WHO STEP wise approach to Surveillance (STEPS) survey in 2013 (3.6%), and previous meta-analyses (8.4% and 8.5%) ^5, 6, 31^. Similarly, the estimated prevalence of pre-diabetes in our study was almost double than what has been reported in other studies ^5, 6^. One explanation for this finding can be the rapid urbanization, and migration from rural to urban areas which has promoted a sedentary lifestyle among individuals, along with consumption of unhealthy foods ^32^. As per our study, high BMI was the main cause of T2DM in Nepal. In South Asia, lifestyle factors such as poor diet, and increased sedentary behaviors with limited physical activities have contributed to the rise of overweight and obesity among children and adolescents ^33^. Rapid development of the economic situation in developing countries like Nepal has resulted in a change of diet rich in cereal and vegetables to one with animal products and processed food with high fat and sugar content ^34^. In a study by Hills *et al.* the prevalence of overweight in Nepal was estimated to be 16.7%, with a higher prevalence in women (19.6%) compared to men (13.6%) ^34^. Obesity is closely linked with premature onset of T2DM and cardiovascular disease ^35^. A similar increasing trend of T2DM led by obesity is seen in Africa as well ^36^. It is important to target T2DM risk factors in order to take control of this disease in Nepal. Physicians advise for special diet and regular exercise for diabetic patients, however, there are noncompliance has been observed in Nepalese population ^37^. Our findings highlight the importance of exercise and a healthy diet to prevent the increased morbidity among individuals with T2DM in this country. Shrestha *et al.* found that the T2DM awareness to be low, with nearly half of the population unaware of the fact that they had this disease ^6^. Increasing public awareness about non-communicable diseases like T2DM and hypertension, and the need to implement a healthy lifestyle is of paramount importance given that our results indicated that individuals with normal blood pressure had less chance of developing T2DM compared to those with hypertension. Increased intake of oily foods, reusing cooking oils which can cause increased conversion of unsaturated fats to trans fats, and low consumption of fruits and vegetables have been found throughout South Asia ^38, 39^. These unhealthy dietary habits lead to increased risks of non-communicable diseases like T2DM and hypertension. Thus, interventions are needed to better manage the overweight and obesity epidemic. This can be achieved through various measures such as opening public parks in the cities for exercise, educating the population about what a healthy lifestyle entails such as decreasing the intake of oily foods, increasing the intake of fruits and vegetable, as well as improving the quality of food. Our study has several strengths. Firstly, we performed comprehensive literature search to pool the results of fifteen studies over the last twenty years to evaluate the prevalence of T2DM in Nepal. In addition, no prior meta-analysis has evaluated the risk factors for T2DM, specifically IGT in Nepal, prior to our study. We also analyzed data based on a time frame, where significant increase in T2DM prevalence was observed in Nepal when comparing 2010–2015 with 2015–2020. Our study had some limitations. There was heterogeneity in the studies due to variation in the T2DM diagnostic criteria, different demographics of the population, etc. Most of the included studies were based on specific areas such as province 1 and 3, and not enough studies have been done on a national scale. Finally, risk factors for T2DM were not reported in all the studies that were included.

## Conclusion

The prevalence of T2DM, pre-diabetes and IGT in Nepal was estimated to be 10%, 19.4% and 11% respectively. Obesity is the major risk factor of T2DM in Nepal and people with normal waist circumference, normal blood pressure and lack of family history of T2DM had lower odds of developing this disease.

## Data availability statement

### Underlying data

Figshare: Diabetes Mellitus in Nepal from 2000 to 2020: A systematic review and meta-analysis

https://doi.org/10.6084/m9.figshare.14706648.v1 ^12^

The project contains the following underlying data:

Dataset: Quantitative data, glycemic control, socio-economic status, BMI, exercise, T2DM prevalence, waist circumference, family history, fruit and vegetable serving per day, alcohol consumption, smoking, education, and BP)

### Extended data

Figshare: Diabetes Mellitus in Nepal from 2000 to 2020: A systematic review and meta-analysis

https://doi.org/10.6084/m9.figshare.14854065.v1 ^10^

The project contains the following underlying data:

- Data file 1: Electronic search details
- Data file 2: Additional analysis
- Data file 3: PRISMA checklist

Data are available under the terms of the Creative Commons Zero "No rights reserved" data waiver (CC0 1.0 Public domain dedication).

## Authors' contributions

DBS, PB, and YRS contributed to the concept and design, analysis, and interpretation of data. DBS, PB, AM, SL, SN, AA, and AP contributed to the literature search, data extraction, review, and initial manuscript drafting. YRS, SN, and AA interpretation of data, revising the manuscript for important intellectual content, and approval of the final manuscript.

All authors were involved in drafting and revising the manuscript and approved the final version.
