# Supplementary material for: Type 2 Diabetes Mellitus in Nepal from 2000 to 2020: A systematic review and meta-analysis
Source: F1000Res. 2021 Sep 6;10:543. Originally published 2021 Jul 7. [Version 2] doi: 10.12688/f1000research.53970.2 (PMC8459622; doi:10.12688/f1000research.53970.2)
Supplement: Peer review history

# Peer review: Reviewer response for version 2

**DOI:** 10.5256/f1000research.77195.r93500

**Published:** 2021-09-22

**Authors:** Sijan Basnet

**Affiliations:** 1 Department of Internal Medicine, Reading Hospital and Medical Centre, West Reading, PA, USA

Thank you for adding limitations to your article.

Are the rationale for, and objectives of, the Systematic Review clearly stated?

Yes

Is the statistical analysis and its interpretation appropriate?

Yes

Are sufficient details of the methods and analysis provided to allow replication by others?

Yes

Are the conclusions drawn adequately supported by the results presented in the review?

Yes

Reviewer Expertise:

Internal Medicine

I confirm that I have read this submission and believe that I have an appropriate level of expertise to confirm that it is of an acceptable scientific standard.

# Peer review: Reviewer response for version 2

**DOI:** 10.5256/f1000research.77195.r93501

**Published:** 2021-09-07

**Authors:** Chudchawal Juntarawijit

**Affiliations:** 1 Department of Natural Resources and Environment, Faculty of Agriculture, Natural Resources, and Environment, Naresuan University, Phitsanulok, Thailand

Although the author has addressed some of my comments, there were still a few important points to be elaborated on before final approval.

- Prevalence of pre-diabetes and T2DM were largely different from those found in previous meta-analysis studies, one recently published in the year 2020. This inconsistency may not be solely explained by the fact that there was a rapid increase in urbanization and lifestyle change as claimed by the authors.
- The main study objective was to identify risk factors. However, information on the issues was very limited, and the study failed to identify some common risk factors, e.g. cigarette smoking, alcohol consumption, fruit and vegetable intake, and exercise. The association between these common risk factors and diabetes has been well supported by various studies from different countries. The discrepancy might cause by bias or the study limitations, e.g. small sample size. To avoid any misleading and faulty conclusions, further discussion and study limitations should be explicitly presented. The discussion section should be reorganized into subsections by the risk factors.
- In the discussion and conclusion, the author stated that obesity was the only major risk factor. However, the relative strength of the contribution of individual factors was not analyzed or adequately discussed before. As mention earlier, this might cause misleading.
- The quality of every figure was still needed to be improved as already suggested.

Are the rationale for, and objectives of, the Systematic Review clearly stated?

Partly

Is the statistical analysis and its interpretation appropriate?

Partly

Are sufficient details of the methods and analysis provided to allow replication by others?

Partly

Are the conclusions drawn adequately supported by the results presented in the review?

Partly

Reviewer Expertise:

Environmental health science

I confirm that I have read this submission and believe that I have an appropriate level of expertise to confirm that it is of an acceptable scientific standard, however I have significant reservations, as outlined above.

# Peer review: Reviewer response for version 1

**DOI:** 10.5256/f1000research.57408.r89248

**Published:** 2021-09-06

**Authors:** Prajwal Gyawali

**Affiliations:** 1 School of Health and Wellbeing, Faculty of Health, Engineering and Sciences, University of Southern Queensland, Toowoomba, QLD, Australia

A) Background

1. Please check for sentence structure;
  *"In 2019, the International Diabetes Federation (IDF) estimated that 463 million adults worldwide had diabetes ^1^. The statistics showed that these individuals were in the age range of 20 to 79, **have diabetes**, and 79.4% were from low- and middle-income countries."*
2. Reference number 4 used in the background does not match with the authors' claim. This reference is more focused on foetal programming contributing to the prevalence of obesity in Nepal.
3. There are already two systematic reviews published in this sector, references 5 and 6:
  Gyawali B, Sharma R, Neupane D, *et al.*: Prevalence of type 2 diabetes in Nepal: a systematic review and meta-analysis from 2000 to 2014 ^1^.
  Shrestha N, Mishra SR, Ghimire S, *et al.*: Burden of Diabetes and Prediabetes in Nepal: A Systematic Review and Meta-Analysis ^2^.
4. Authors need to provide a valid reason for conducting this systematic review in the same area in the background section. Paper by Shrestha N. *et al.* ^2^ was published less than 1 year ago and they have included 14 papers for the final analysis.

B) Methods

1. The authors have reported the prevalence of impaired glucose tolerance and prediabetes. Why were these keywords not used in the search strategy? Likewise, have the authors used the terminology 'glucose' as a keyword in a search strategy?

C) Discussion

1. The metanalysis published in 2020 by Shrestha N. *et al.* ^2^ included 14 papers in the final analysis. All papers were after 2000. They reported the prevalence of pre-diabetes is much less than what the current paper has reported. The reason provided by the authors for this large difference is not properly addressed in the discussion. This is very important to address. 
2. The papers should be discussed in sub-sections. 

Are the rationale for, and objectives of, the Systematic Review clearly stated?

No

Is the statistical analysis and its interpretation appropriate?

I cannot comment. A qualified statistician is required.

Are sufficient details of the methods and analysis provided to allow replication by others?

No

Are the conclusions drawn adequately supported by the results presented in the review?

Partly

Reviewer Expertise:

Stroke

I confirm that I have read this submission and believe that I have an appropriate level of expertise to confirm that it is of an acceptable scientific standard, however I have significant reservations, as outlined above.

## References

[1] : Prevalence of type 2 diabetes in Nepal: a systematic review and meta-analysis from 2000 to 2014.*Glob Health Action*.2015;8: 10.3402/gha.v8.290882908810.3402/gha.v8.2908826613684PMC4662667

[2] : Burden of Diabetes and Prediabetes in Nepal: A Systematic Review and Meta-Analysis.*Diabetes Ther*.2020;11(9) : 10.1007/s13300-020-00884-01935-194610.1007/s13300-020-00884-032712902PMC7434818

# Peer review: Reviewer response for version 1

**DOI:** 10.5256/f1000research.57408.r91625

**Published:** 2021-08-13

**Authors:** Chudchawal Juntarawijit

**Affiliations:** 1 Department of Natural Resources and Environment, Faculty of Agriculture, Natural Resources, and Environment, Naresuan University, Phitsanulok, Thailand

**Background:**

1. In the introduction, more information about the prevalence and risk factors of diabetes should be presented and reorganized for easy reading. The research problem and hypothesis also need to be clearly articulated.
2. The rationale for the review was also not clearly described in the context of what is already known. As there have been few studies in Nepal, it was not a good reason for conducting meta-analysis. Doing a meta-analysis using a small number of studies might cause bias. 

**Methods:**

1. Study methods were poorly described and hard to read (see a good example study by Miller *et al.* ^1^). To help readers replicate the study, the authors should provide more detailed information on electronic search strategy, methods of results synthesis, and sensitivity analysis.
2. Using only the global search engine, e.g. PubMed, Embase, Scopus, and Google Scholar might cause publication bias if there were some researches papers published locally or in other databases. In point had not been mention in the study limitation.

**Results:**

1. Results were inadequately described. In the qualitative summary, the authors should talk about the important features of the information in Table 1. In quantitative synthesis, information on how the pooled prevalence was calculated is also needed. In every figure, the total events should be indicated, and the figure legend should contain more information.

**Discussion:**

1. In the Discussion, more information should be provided and reorganized for an easy read.
2. In the study limitation, the authors mention T2DM diagnostic criteria and demographic data as a problem. However, the authors did not mention how the problems affect the results and how the study handled them.
3. The statement: “Our finds highlight the importance of exercise and a healthy diet to prevent the increased morbidity among individuals with T2DM in this country”, needs more justification. Was the information from the results of this study or from the literature?
4. In an attempt to identify diabetes risk factors, the study limited only those studies from Nepal. How this will affect the study results?

 

**Conclusion:**

1.  In the conclusion statement, only obesity was claimed to cause diabetes. This conclusion might need more justification since obesity and other risk factors were not extensively discussed. This result was also in contrast to that observed by Jayawardena *et al*. ^2^, a similar study using data from all countries in South Asia. In that study, family history, urban residency, age, higher BMI, sedentary lifestyle, hypertension, and waist-hip ratio were found to be associated with an increased risk of diabetes.
2. The conclusion statement should also provide study limitations and recommendations for public health use.

Are the rationale for, and objectives of, the Systematic Review clearly stated?

Partly

Is the statistical analysis and its interpretation appropriate?

Partly

Are sufficient details of the methods and analysis provided to allow replication by others?

Partly

Are the conclusions drawn adequately supported by the results presented in the review?

Partly

Reviewer Expertise:

Environmental health science

I confirm that I have read this submission and believe that I have an appropriate level of expertise to confirm that it is of an acceptable scientific standard, however I have significant reservations, as outlined above.

## References

[1] : Aerobic, resistance, and mind-body exercise are equivalent to mitigate symptoms of depression in older adults: A systematic review and network meta-analysis of randomised controlled trials. *F1000Research*.2021;9: 10.12688/f1000research.27123.210.12688/f1000research.27123.2PMC819152034158928

[2] : Prevalence and trends of the diabetes epidemic in South Asia: a systematic review and meta-analysis.*BMC Public Health*.2012;12: 10.1186/1471-2458-12-38038010.1186/1471-2458-12-38022630043PMC3447674

# Response

**Published:** 2021-08-29

**Authors:** Alok Atreya

**Affiliations:** Lumbini Medical College, Nepal

Background: In the introduction, more information about the prevalence and risk factors of diabetes should be presented and reorganized for easy reading. The research problem and hypothesis also need to be clearly articulated.

**Reply: Thank you for the comment. We have presented the situation of T2DM in a funnel pattern in the background giving an overview of global, regional, and national scenarios. We have rephrased and edited as per requirement by the reviewer’s comment to further clarify the rationale.**

 The rationale for the review was also not clearly described in the context of what is already known. As there have been few studies in Nepal, it was not a good reason for conducting meta-analysis. Doing a meta-analysis using a small number of studies might cause bias. 

**Reply: Thank you for the comment. We have edited the rationale as per requirement by the reviewer’s comment to further clarify the rationale. We included 15 prevalence-based studies with justifiable sample sizes and assessed the quality of those individual studies using the JBI bias tool to reduce the bias of including studies. Due to the urge of the evidence-based practice, we authors think pooling of the data from available individual studies to give pooled prevalence is justifiable.**

 Methods: Study methods were poorly described and hard to read (see a good example study by Miller et al.1). To help readers replicate the study, the authors should provide more detailed information on electronic search strategy, methods of results synthesis, and sensitivity analysis.

**Reply: Thank you for the comment. We have conducted our study as per the standard guideline and abide by the MOOSE checklist for the meta-analysis of observational studies. Our electronic search details including the link, and additional sensitivity analysis are available in the extended data file and shared publicly.**

 Using only the global search engine, e.g. PubMed, Embase, Scopus, and Google Scholar might cause publication bias if there were some researches papers published locally or in other databases. In point had not been mention in the study limitation.

**Reply: Thank you for the comment. We have conducted our study as per the standard guideline and abide by the MOOSE checklist for the meta-analysis of observational studies. In Nepal, almost all Nepalese local journals are listed in NEPJOL (Nepalese Journal Online), which is the common platform and all the published papers are available through Google scholar so we included the Google scholar database in our review, which is missed by some prior reviews. So, we authors believe including Google scholar and other standard global databases won’t miss published literature.**

 Results: Results were inadequately described. In the qualitative summary, the authors should talk about the important features of the information in Table 1. In quantitative synthesis, information on how the pooled prevalence was calculated is also needed. In every figure, the total events should be indicated, and the figure legend should contain more information.

**Reply: Thank you for the comment. We elaborated qualitative summary as “A qualitative summary of all 15 individual studies is presented in (Table 2). Nine studies were done in a community setting while the rest six were done in the hospital setting. Two community-based studies and one hospital-based study included samples from different parts of Nepal to represent the country, while the rest were loco-regional studies.” For transparency of our work we made our data available in public data repository Figshare; link: **https://doi.org/10.6084/m9.figshare.14706648.v1.

**Regarding data pooling methods, based on heterogeneity we used a random or fixed-effect model. And we mentioned the model used in every section before the description of the individual results and forest plot. Regarding total events, I agree with the reviewer, however, due to the default setting of the software CMA-3, we could not edit the figure.**

 Discussion:

 In the Discussion, more information should be provided and reorganized for an easy read. In the study limitation, the authors mention T2DM diagnostic criteria and demographic data as a problem. However, the authors did not mention how the problems affect the results and how the study handled them. The statement: “Our finds highlight the importance of exercise and a healthy diet to prevent the increased morbidity among individuals with T2DM in this country”, needs more justification. Was the information from the results of this study or from the literature?

**Reply: Thank you for the comment. There are perceived barriers for the non-compliance for physician’s advice to a special diet and regular exercise as shown in a Nepalese study. We have added a reference to justify this statement.**

 In an attempt to identify diabetes risk factors, the study limited only those studies from Nepal. How this will affect the study results?

**Reply: Thank you for the comment. Diabetes prevalence and risk differ across different societies and regions across the globe. This study is solely aimed to estimate prevalence and risk factors in the context of Nepal to build foundation evidence to make evidence-based practice in Nepal so may be different than other regions across the globe.**

 Conclusion:  In the conclusion statement, only obesity was claimed to cause diabetes. This conclusion might need more justification since obesity and other risk factors were not extensively discussed. This result was also in contrast to that observed by Jayawardena et al. 2, a similar study using data from all countries in South Asia. In that study, family history, urban residency, age, higher BMI, sedentary lifestyle, hypertension, and waist-hip ratio were found to be associated with an increased risk of diabetes. The conclusion statement should also provide study limitations and recommendations for public health use.

**Reply: Thank you for the comment. The conclusion statement states obesity as one of the many major risk factors for diabetes. We kindly apologize, but we have not claimed only obesity causes diabetes. The limitations have already been provided at the end of the discussion section, so due to fear of duplication, we did not include them in the conclusion section.**

# Peer review: Reviewer response for version 1

**DOI:** 10.5256/f1000research.57408.r89249

**Published:** 2021-07-29

**Authors:** Sijan Basnet

**Affiliations:** 1 Department of Internal Medicine, Reading Hospital and Medical Centre, West Reading, PA, USA

This is a very well-written manuscript with robust statistical analysis. 

1. Please mention the limitations of your study if any.
2. Most of the studies cited are from the 2010s. There is one from 2000 with 1840 subjects from an outpatient clinic in a city that may not be representative of the country’s demographics and one from 2006 with 1012 subjects from seven wards that are being used to study the trend. Does this skew your results in any way? Also, the authors justify the increasing prevalence of rapid urbanization but the above studies were done in cities. 

Are the rationale for, and objectives of, the Systematic Review clearly stated?

Yes

Is the statistical analysis and its interpretation appropriate?

Yes

Are sufficient details of the methods and analysis provided to allow replication by others?

Yes

Are the conclusions drawn adequately supported by the results presented in the review?

Yes

Reviewer Expertise:

Internal Medicine

I confirm that I have read this submission and believe that I have an appropriate level of expertise to confirm that it is of an acceptable scientific standard, however I have significant reservations, as outlined above.

# Response

**Published:** 2021-08-29

**Authors:** Alok Atreya

**Affiliations:** Lumbini Medical College, Nepal

Thank you for the comment. Due to the relatively few studies from 2000-2010. We did an analysis based on a published study between 2010-2015, and 2015-2020 as “The assessment of T2DM prevalence between 2010–2015 with the use of random-effects meta-analysis was 7.75% (Proportion, 0.0775; 95% CI, 0.0367-0.1561; studies: 4; I ^2^:99.62), while this value increased to 11.24%, between 2015–2020 (Proportion, 0.1124; 95% CI, 0.0789-0.1577; I ^2^: 96.74)”. Analysis showed some increasing trends of T2DM in Nepal which is of concern.

 Being our meta-analysis is a secondary analysis of the published literature, we do have some limitations due to primary studies variation. We have included our study limitations as “Our study had some limitations. There was heterogeneity in the studies due to variation in the T2DM diagnostic criteria, different demographics of the population, etc. Most of the included studies were based on specific areas such as provinces 1 and 3, and not enough studies have been done on a national scale. Finally, risk factors for T2DM were not reported in all the studies that were included.”
